# Dysmenorrhoea among students aged 18–45 years attending University in Uganda: A cross‐sectional multicenter study of three Universities in Uganda

**DOI:** 10.1002/nop2.207

**Published:** 2018-09-27

**Authors:** Rose Mary Nakame, Frank Kiwanuka, Afayo Robert

**Affiliations:** ^1^ Irise Uganda, YALI East Africa Alumni Gender Equality Task Force Research‐Group National Menstrual Steering Committee Kampala Uganda; ^2^ Department of Critical Care Nursing Tehran University of Medical Sciences School of Nursing Tehran Iran; ^3^ Clarke International University School of Nursing Kampala Uganda

**Keywords:** Africa, dysmenorrhoea, students, Uganda, university

## Abstract

**Aim:**

Dysmenorrhoea presents as pain associated with menstruation. It is often an issue discussed privately, yet it continues to affect girls and women with grave impact on their education, social activities and work. This study sought to assess the factors associated with dysmenorrhoea among female students aged 18–45 years in three selected universities in Kampala Capital city, Uganda.

**Design and Methods:**

The study was a cross‐sectional study conducted among 351 female students of three Universities in Kampala, Uganda. Purposive sampling methods were used. A self‐administered questionnaire was used for data collection, a 95% confidence interval was considered and analysis was done using SPSS version 20.

**Results:**

Respondents (*N* = 351) fully completed the study out of 383 invited participants. The prevalence of dysmenorrhoea was 75.8%. Associated factors included having children (*p* < 0.05), daily consumption of sugary foods (*p* < 0.05) and family history of dysmenorrhoea (*p* < 0.01).

**Conclusion:**

As nursing professionals, we believe comfort is a fundamental determinant of health. Therefore, evidence suggesting a high prevalence of dysmenorrhoea as revealed in this study is concerning. We recommend schools to have school clinics and school nurses who could help students during such days of discomfort. Further studies assessing the casual relationships of various correlates to dysmenorrhoea and the impact of dysmenorrhoea specifically on academic life of the students should be conducted.

## 1 INTRODUCTION

Menstruation is a natural phenomenon in females. Menstruation disorders like dysmenorrhoea affect the life of women. Dysmenorrhoea is a critical public health burden owing to its high prevalence across studies. Globally, the prevalence of dysmenorrhoea varies from 20% to 90% (De Sanctis et al., 2015). In Europe and America, the prevalence of dysmenorrhoea has been reported to range from 52.4% to 85.7% (Grandi, Serena, & Angelo, 2012; Kaunitz & Smith, 2014).

The prevalence of dysmenorrhoea in Asia ranges from 58.8% to 84.9% (Chan, 2009; Mahkam, Joffres, Corber, Bayanzadeh, & Mahnaz, 2011). Various studies in Africa have reported that the prevalence ranging from 58% to 85.4% (Aziato, Dedey, & Clegg‐Lamptey, 2014; Nooh, 2014). However, there is paucity of evidence available about the prevalence of dysmenorrhoea in East Africa where Uganda is located.

Various studies have reported that dysmenorrhoea is associated with age, marital status, parity, and body mass index and null parity and duration of flow (De Sanctis et al., 2015; Faramarzi & Salmalian, 2014). Mean age at menarche, family history and irregular periods increase the occurrence of dysmenorrhoea (Sznajder, Harlow, Burgard, Wang, & Han, 2014). A similar study reported that a woman would be more likely to have dysmenorrhoea if she had a positive family history for dysmenorrhoea (Tinatin, Besarion, & Gagua, 2012). Furthermore, a high job strain and dysmenorrhoea were found to be significantly associated in another study (Jung & Lee, 2013; Sznajder et al., 2014).

Lifestyle characteristics such as alcohol consumption, coffee consumption, sugar intake, smoking and physical activity have also been associated with dysmenorrhoea (Helwa, Mitaeb, Al‐Hamshri, & Sweileh, 2018; Tinatin et al., 2012). Consequences of dysmenorrhoea include altered concentration in class (Akinnubi, 2016), absenteeism, emotional disturbances and altered sleep patterns (Michiko, Momoeda, Kubota, & Nakabayashi, 2011). Notwithstanding, the girl child’s welfare is the responsibility of the family, government and the community where she thrives. This implies the parents or even the whole family, the school matrons, teachers and school nurses have an important role in the issues concerning the girls like menstruation. This said, the African tradition also considers issues surrounding menstruation as secretive issues which the girl child should discuss in privacy with her parents or senior women in the community (Aziato et al., 2014). Consequently, girls at schools continue to bear the pain “till when they deliver a child” a saying they have been told by their matrons, and in their communities since the first occurrence of dysmenorrhoea. While based on anecdotal evidence, the school matrons have made the students believe that one should tolerate dysmenorrhoea after all the labour pain is worse. Owing to the above phenomenon, this study sought to determine the prevalence of dysmenorrhoea, and correlated factors among students aged 18–45 years in three selected universities in Kampala, Uganda.

## 2 METHODS

### 2.1 Study design and setting

A cross‐sectional study was conducted in three institutions in Kampala namely: Kampala University (KU) Mutundwe campus; International Health Sciences University (IHSU); and Mulago School of Nursing, Kampala, Uganda.

### 2.2 Study population and sampling procedure

#### 2.2.1 Inclusion and exclusion criteria

Female students registered with the institutions during the period of the study aged 18–45 years were eligible to participate in the study. Female students with other chronic gynaecologic conditions were excluded from the study. The researchers estimated a sample size of 383 participants.

#### 2.2.2 Sampling technique

Purposive sampling method was used to select the institutions and students who took part in the study. Sample size proportionate to population size was considered in each setting. To estimate the study sample size, we adopted 85.4% prevalence (Fatai, Nonyelum, Suleman, Adeniyi, & Okoye, 2013), a significance level of 95%, an accepted error of 0.05 and *a* design effect (*Deff *≥ 1) for cluster sampling since the students in each institution or class are similar. We then used the Kish (Kish, 1965) formula to estimate the sample and came up with an estimated sample size of 383 participants.

### 2.3 Data collection tool

A structured self‐administered questionnaire was developed by the researchers from literature review. The instrument assessed demographic data, the prevalence of dysmenorrhoea, lifestyle characteristics of the participants. Guidelines of the Society of Obstetrics and Gynecologists of Canada (SOGC, 2005) were used to elicit menstrual history. The questionnaire was pretested before dissemination. The pre‐test exercise was carried out among 20 students at International Health Sciences University. Adjustments were done to the tool after the pre‐test exercise. Content validation of the tool was done by four professionals at IHSU. The questionnaire took approximately 10 min to complete.

### 2.4 Data collection procedure

After obtaining ethical clearance from the relevant authorities, the researcher published notices on the notice boards of respective institutions inviting eligible students to participate in the study. The students’ Guild Leaders were also used to spread information about the study. Data were collected from eligible participants during class breaks using a self‐administered questionnaire.

The data collection process took place at the selected campuses during class breaks. The questionnaire was self‐administered. The questionnaire was completed in the presence of a research assistant to elaborate on any issues that needed clarity. The data collection was conducted for 4 months from May‐July 2015. Research assistants were trained vigorously prior to collection of data.

### 2.5 Data management and analysis plan

Completed questionnaires were cross‐checked for completeness immediately after they were received from the respondents, and data were then entered into Epi‐info version 7. Here, check codes were used to minimize errors that could occur during data entry and to speed up the data entry process. Data were then exported and analyzed in Statistical Packages for Social Scientists (SPSS) version 20.

Descriptive information on respondents’ characteristics was presented in form of frequency tables. Descriptive analysis provided measures of central tendency and dispersion for numerical variables. A level of significance of 0.05 was considered significant in this study.

#### 2.5.1 Measuring dysmenorrhoea and its correlates

Experience of dysmenorrhoea was dichotomized in “Yes” and “No,” and a provision for none respondents was provided in the questionnaire owing to the sensitivity of this question. The experience of dysmenorrhoea was elicited considering up to the four previous menstrual cycles.

Inferential statistics were performed to assess the correlates to dysmenorrhoea using chi‐squared test, Analysis of Variance (ANOVA) and other statistical tests were considered where appropriate. Variables that were significant at bivariate analysis were further analyzed using multivariate analysis to ascertain independent variables correlated to dysmenorrhoea.

### 2.6 Ethical and legal considerations

Research and ethics committee of IHSU approved the research protocol. Further administrative clearance was sought from the administrators of KU and Mulago School of nursing to carry out the study on their premises. The aim of the study was clearly explained to the participants and written consent forms were availed before issuing the questionnaire. Anonymity was maintained at times to encourage the participants to share their private information with us.

Anonymity was assured by labelling the questionnaires with serial numbers instead of the names of participants. Considering that the participants were not under any oath to share the truth, consistency of the data was checked prior to data entry.

## 3 RESULTS

The 351 respondents fully completed the study out of 383 invited participants, making a 91.6% response rate.

### 3.1 Socio‐demographic characteristics

The mean age of the respondents was 22 years (*SD* 1.4). The majority (81.8%) of the respondents’ age ranged from 18 to 24 years with only 1.7% of respondents aged between 39 and 45 years (Table 1). Most respondents were single (81.8%), had not yet given birth to a child (75.8%), unemployed (82.1%) and Catholic (33.9%) with regard to their religious domain (Table 1).

**Table 1:** Socio‐demographic characteristics among female students in 3 institutions of higher learning in Kampala (*N* = 351)

Characteristics	Attributes	Frequency (*N*)	Percentage (%)
Age (in years)	18–24	287	81.8
	25–31	51	14.5
	32–38	7	2.0
	39–45	6	1.7
Marital status	Single	287	81.8
	Married/Cohabiting	51	14.5
	Widowed	7	2.0
	Divorced/Separated	6	1.7
Number of children	None	266	75.8
	≤4	79	22.5
	≥4	6	1.7
Employment status	Employed	63	17.9
	Unemployed	288	82.1
Religion	Protestant	73	20.8
	Catholic	119	33.9
	Muslim	49	14.0
	Pentecostal	89	25.4
	SDA	16	4.6
	Other	5	1.4

### 3.2 Lifestyle characteristics of the respondents

Majority (88%) of the respondents did not consume alcohol (Table 2). Of the 42 respondents who consumed alcohol, more than half 27 (64.3%) consumed 1–2 servings of alcohol per week with an average consumption of 1.74 servings (*SD* 1.106). Most respondents (60.4%) consumed coffee, out of these 92.9% drunk 1–3 cups of coffee per day. Most (92.9%) female students consumed sugar while as 82.3% consumed sugar in tea. Majority (96.6%) did not smoke cigarettes or shisha (Table 2). A total of 181 (51.6%) female students exercised regularly, of these, most of the respondents were walking while others ran, skipped a rope, jogging, aerobics, sit‐ups, jumping up, swimming, volleyball, netball, pressure ups and running (Table 2).

**Table 2:** Lifestyle characteristics of female students aged 18–45 years in 3 institutions of higher learning in Kampala (*N* = 351)

Characteristics	Attributes	Frequency (*N*)	Percentage (%)
Alcohol Consumption	Yes	42	12.0
	No	309	88.0
Alcohol servings per week	1–2	27	64.3
	3–4	4	9.5
	5–6	6	14.3
	>7	5	11.9
Coffee consumption	Yes	212	60.4
	No	139	39.6
Cups of coffee consumed per day	1–3	197	92.9
	4–6	9	4.2
	7–9	5	2.4
	>10	1	0.5
Sugar consumption	Yes	326	92.9
	No	25	7.1
Daily consumption of sugary foods	Sugar in tea	289	82.3
	Ice cream	11	3.1
	Chocolate	5	1.4
	Sweets	14	4.0
	All of them	12	3.4
	None	20	5.7
Smoking of cigarettes or shisha	Yes	12	3.4
	No	339	96.6
Regular exercise	Yes	181	51.6
	No	170	48.4
Times per week of exercise (days)	1	33	18.2
	2–3	63	34.8
	4–5	31	17.1
	6–7	54	29.8
Duration of exercise (minutes)	≤30	87	48.1
	>30	94	51.9

With regard to exercise, a total of 34.8% (63) female students exercised 2–3 times per week, an average of 2.59 times (*SD* 1.100). A total of 51.9% (94) spent more than 30 min while exercising as shown in Table 2.

### 3.3 Prevalence of dysmenorrhoea and menstrual‐related characteristics of the respondents

Most 75.8% (266) of the respondents reported that they had ever experienced dysmenorrhoea with an average duration of 1.91 (*SD* 0.793). Of the respondents, 41% reported that they often experience dysmenorrhoea for 1–2 days.

Most respondents (75.2%) reported that experiencing dysmenorrhoea affects their concentration in class while 20.3% reported that it had no effect on their classroom concentration (Table 3).

**Table 3:** Dysmenorrhoea among the respondents

Characteristics	Attributes	Frequency (*N*)	Percentage (%)
Ever experienced dysmenorrhoea in the last 4 menstrual cycles	Yes	266	75.8
	No	70	20
	None respondents	15	4.2
Duration of experiencing dysmenorrhoea (*N* = 266)***	1–2 days	109	41
	3–4 days	68	25.6
	>4 days	11	4.1
	None respondents	78	29.3
Experience of menstruation affects your concentration in class (*N* = 266)***	Agree	200	75.2
	Disagree	54	20.3
	Nonrespondents	12	4.5
Age at menarche	8–11	12	3.4
	12–15	292	83.2
	16–19	47	13.4
Experience monthly period	Yes	325	92.6
	No	26	7.4
Length of menstrual period (days)	1–2	22	6.3
	3–4	256	72.9
	5–6	61	17.4
	>7	12	3.4
Taking drugs for any long‐term illness	Yes	7	2.0
	No	344	98.0
Pain reliever	Painkillers	130	48.9
	Rest	82	30.8
	Mineral supplements	3	1.1
	Warm water	28	10.5
	Contraceptives	2	0.8
	Herbs	2	0.8
	Others	19	7.1
Possession of relatives with dysmenorrhoea	Yes	205	58.4
	No	146	41.6
Relative with dysmenorrhoea	Mother	18	8.78
	Sister	127	61.95
	Cousin	45	21.95
	Other	15	7.32
Work/institution related stress	Yes	241	68.7
	No	110	31.3
Rating of work/institution related stress	Low	48	19.9
	Moderate	160	66.4
	Severe/very severe	33	13.7
Age at menarche	8–11	12	3.4
	12–15	292	83.2
	16–19	47	13.4
Experience monthly period	Yes	325	92.6
	No	26	7.4

***Only those that experienced dysmenorrhea in the last 4 menstrual cycles.

The average age at menarche for the respondents was 13 years (*SD* 1.498), ranging from 9‐19 years) with most 83.2% girls’ age at menarche falling in between 12–15 years. Most (92.6%) of the respondents experienced their periods every month, 58.4% had a relative with dysmenorrhoea, with 61.95% of the respondents citing a sister as the person experiencing dysmenorrhoea (Table 3).

Other consequences of dysmenorrhoea reported included: work‐related stress reported by more than half (68.7%) of the respondents, of which 66.4% reported moderate level of stress. Only 2% of the respondents were taking drugs for a long‐term illness, which consisted of anti‐hypertensives, contraceptives, antibiotics, and broncho‐dilators as the drugs being used while 48.9% used painkillers (Table 4). Most (72.9%) of the female students had their monthly menstrual period lasting 3–4 days with a mean length of the monthly period lasting 2.18 days (*SD* 0.585).

**Table 4:** Factors associated with the prevalence of dysmenorrhoea at multivariate analysis

Variable	Category	OR (95% CI)	*p*‐Value
Number of children	None	1	
	1–4	0.35 (0.192–0.641)	0.001
	>4	0.128 (0.021–0.767)	0.024
Possession of a relative with dysmenorrhoea	Yes	1	
	No	2.583 (1.509–4.423)	0.001
Daily consumption of sugary foods	Sugar in tea	1	
	Chocolate	0.09 (0.014–0.571)	0.011
	None	0.344 (0.130–0.911)	0.032

*At multivariate analysis;* having children (*p* < 0.05), taking chocolate (OR = 0.09, *p* = 0.011) or no sugary foods at all (OR = 0.344, *p* = 0.032) were protective against the occurrence of dysmenorrhoea while respondents who did not possess a relative with dysmenorrhoea were 2.583 times more likely to experience dysmenorrhoea (Table 4).

## 4 DISCUSSION

In this study, we sought to determine the prevalence of dysmenorrhoea and its correlates among female students. The prevalence of dysmenorrhoea found among the three institutions in Kampala was high with three‐quarters of students experiencing dysmenorrhoea. The socio‐demographic characteristics of our sample are quite similar to most institutions of higher learning in Uganda. This could imply that most female students in Universities in Uganda experience some form of dysmenorrhoea. Based on this finding, we recommend schools to have nursing services at the school premises. These could help students to manage dysmenorrhoea.

The prevalence of dysmenorrhoea in this study is congruent with that reported in similar studies in Africa that reported a prevalence ranging from 58% to 85.1% (Nooh, 2014; Ogufowokan & Babatunde, 2010; Shiferaw, Wubshet, & Tegabu, 2014). Consequences of dysmenorrhoea reported included: effect on concentration in class and work‐related stress. Altered concentration in class could lead to absenteeism and inattentiveness in class which led to poor grades in class (Aziato et al., 2014).

Similar studies have reported similar discomforts including physical fatigue and emotional instability manifested as nervousness/irritability (Helwa et al., 2018). Our study reported on the effect of reduced concentration in class; however, its association with classroom grades was beyond our scope. Future studies could investigate whether dysmenorrhoea among female students affects their academic performance in Uganda.

Our findings with regard to the prevalence of dysmenorrhoea were slightly higher than the 65.4% prevalence reported in Egypt by Nooh, (2014). This disparity could be attributed to differences in the definition of dysmenorrhoea used across studies owing to lack of a standard method for assessing the prevalence of dysmenorrhoea.

Correlates to dysmenorrhoea included possession of children, having a relative with dysmenorrhoea and daily consumption of sugary foods. Consumption of chocolate or no consumption of sugary foods at all was protective against the occurrence of dysmenorrhoea. This could be due to the polyphenols in dark chocolate that could improve the circulation of blood by dilating the arteries and increase nitric oxide.

A reduced sugar intake for 7–10 days prior the onset of menses has been linked to a decrease in fluid retention thus decreasing systemic effects related to dysmenorrhoea (Lowdermilk, Shannon, & Mary, 2013). The sugary foods that were asked about were ice cream, chocolate, sweets and sugar in tea. These discoveries agreed with what other scholars (Ozerdogan, Sayiner, Ayranci, Unsal, & Giray, 2009; Tinatin et al., 2012) found in Georgia and Turkey.

This would imply that increased sugar intake could have a range of health problems; it could predispose one to dysmenorrhoea. However, since our study was a cross‐sectional study, we could not infer much on the causal effect of consumption of sugary foods and dysmenorrhoea. The findings on the association between the consumption of sugary foods differed from those inferred in Helwa and colleagues’ study (2018). Further discussion on the causal relationship between sugary foods, having children and relative with dysmenorrhoea is beyond the scope of this study owing to the nature of the study design. Therefore, we recommend further studies on these issues.

In the context of alcohol consumption, a considerably low number of respondents reported consuming alcohol in this study. This could be attributed to the fact that two of the institutions (KU & Mulago School of Nursing) provide accommodation for their student and alcohol is not accepted on the school premises. Indeed, there was no significant relationship between alcohol consumption and dysmenorrhoea. However, Mahkam et al. (2011) study in Iran revealed that having seven servings of alcohol per week decreased the experience of undesirable menstrual pain.

This study found no significant relationship between coffee consumption or the amount of coffee consumed per day and dysmenorrhoea. This finding was consistent with that of Nnaemeka, Malgwi, and Okoro (2013) study among 320 university students in Maidugiri, Nigeria. On the other hand, our results differed from those of Jones, Hong, and Gita (2014) study among 9,067 young Australian women and those reported in Seven, Güvenç, Akyüz, and Eski (2014) study among 380 Turkish nursing students.

Owing to the negativity of smoking especially among women, few respondents in our study reported being smokers of cigarettes or Shisha. This could also be attributed to the prohibition of smoking in public in Uganda. Nonetheless, statistical analysis revealed no association between smoking and dysmenorrhoea in this study.

Even though many respondents reported to participate in at least a particular exercise, we did not establish a significant association between exercise and dysmenorrhoea its frequency and duration with dysmenorrhoea. These results have been reported elsewhere in other studies of scholars in Nigeria and Italy (De Sanctis et al., 2014; Fatai et al., 2013). However, our findings contrasted Vaziri et al. (2015) randomized clinical trial report of physical activity reducing the severity of dysmenorrhoea among 105 students in Iran. This difference in results may be explained by the difference in study designs employed among the study population.

We recommend a randomized clinical trial among the female students in Uganda to explore this relationship between physical activity and exercise. Family history was the only statistically significant variable of all the personal characteristics studied among respondents. Owing to the cross‐sectional nature of the study design, we cannot infer about the causal relationship of familial history and dysmenorrhoea. However, there could be a linkage between these two notions owing to the similarity in the genetic make‐up of close relatives. Therefore, this could predispose one with a family member with dysmenorrhoea to a higher risk of experiencing dysmenorrhoea.

In fact, multivariate analysis revealed an association between family history of dysmenorrhoea and experience of dysmenorrhoea among the respondents. This outcome is contradictory with previous studies that have found respondents with a relative experiencing dysmenorrhoea more susceptible to its occurrence (Mahkam et al., 2011; Seven et al., 2014; Tinatin et al., 2012).

Seven in ten of the respondents experienced their monthly periods for 3–4 days which is normal considering that the most females experience menses for 2–7 days. We did not find any statistical relationship between the duration of menses and the occurrence of dysmenorrhoea. However, we did not find any statistically significant link between dysmenorrhoea and menstrual regularity. These results are congruent with those of Badria, Manal, Latifa, and Samar (2014) study among 924 university students in Saudi Arabia though contrary to those of Sznajder, Harlow, Burgard, Wang, and Han (2014) reported in Tianjin, China.

Our study identified no statistically significant relationship between being stressed, its grade with the occurrence of dysmenorrhoea. Three‐fifths of the respondents reported stress at work or institution which was moderate in nature. This result was in contrast with other scholars’ findings (Kordi, Soheila, & Taghi, 2013; Sznajder et al., 2014).

The relationship between age at menarche and dysmenorrhoea was statistically insignificant. This result was contrary to Nooh’s (2014), report of a dysmenorrhoea among 297 students in Egypt.

This disparity in outcomes may be enlightened by the difference in study location, the definition of dysmenorrhoea used and the difference in lifestyle characteristics of the respondents.

### 4.1 Limitations of the study

This study used non‐probability sampling methods which are inherent to selection bias. We could not infer on the causal relationship between intake of sugary foods and dysmenorrhoea owing to the design of our study.

## 5 CONCLUSION

There is a high prevalence of dysmenorrhoea among female students in Universities. The presence of dysmenorrhoea despite the use of analgesics among students could be a strong pointer to discrepancies in grades and concentration in class among female students compared with their male counterparts. We recommend schools to have school nurses who could help students during these days of discomfort. In addition, health education on causes, management and the need for school clinics to stock analgesics. Further studies should be conducted to determine casual relationships and the impact of dysmenorrhoea on specifically on the academic life of the students. The novelty of these finding is of particular importance for the profession of nursing, as we are well situated to influence these social aspects.

## CONFLICT OF INTEREST

The authors declare no conflict of interest.

## AUTHOR CONTRIBUTIONS

RMN contributed to data collection and ethical clearance for this project while all authors contributed to curation, methodology, literature review, data analysis, and writing of the final Manuscript.
